# Pacing Hippocampal Sharp-Wave Ripples With Weak Electric Stimulation

**DOI:** 10.3389/fnins.2018.00164

**Published:** 2018-03-15

**Authors:** Huiyi Jiang, Shicheng Liu, Xinling Geng, Adam Caccavano, Katherine Conant, Stefano Vicini, Jianyoung Wu

**Affiliations:** ^1^Department of Pediatrics, The First Hospital of Jilin University, Chang Chun, China; ^2^Department of Neuroscience, Georgetown University Medical Center, Georgetown University, Washington, DC, United States; ^3^School of Biomedical Engineering, Capital Medical University, Beijing, China; ^4^Department of Pharmacology, Georgetown University Medical Center, Georgetown University, Washington, DC, United States

**Keywords:** Sharp wave-ripples, pacing, electrical stimulus, hippocampus, CA3, CA1, mouse

## Abstract

Sharp-wave ripples (SWRs) are spontaneous neuronal population events that occur in the hippocampus during sleep and quiet restfulness, and are thought to play a critical role in the consolidation of episodic memory. SWRs occur at a rate of 30–200 events per minute. Their overall abundance may, however, be reduced with aging and neurodegenerative disease. Here we report that the abundance of SWR within murine hippocampal slices can be increased by paced administration of a weak electrical stimulus, especially when the spontaneously occurring rate is low or compromised. Resultant SWRs have large variations in amplitude and ripple patterns, which are morphologically indistinguishable from those of spontaneous SWRs, despite identical stimulus parameters which presumably activate the same CA3 neurons surrounding the electrode. The stimulus intensity for reliably pacing SWRs is weaker than that required for inducing detectable evoked field potentials in CA1. Moreover, repetitive ~1 Hz stimuli with low intensity can reliably evoke thousands of SWRs without detectable LTD or “habituation.” Our results suggest that weak stimuli may facilitate the spontaneous emergence of SWRs without significantly altering their characteristics. Pacing SWRs with weak electric stimuli could potentially be useful for restoring their abundance in the damaged hippocampus.

## Introduction

Sharp-wave ripples (SWRs) are spontaneous neuronal population events that occur in the hippocampus during sleep and quiet restfulness (John and Lynn, 1979; O'keefe and Nadel, 1979; Buzsáki et al., 1983; Axmacher et al., 2008; Buzsáki, 2015). An experience, such as field exploration, is registered in the hippocampus as sequential activations of neuronal assemblies (aka “place cell assemblies”). These sequences are then re-activated during SWRs in off-line states (Wilson and McNaughton, 1994; Lee and Wilson, 2002; Ji and Wilson, 2007; Dragoi and Tonegawa, 2011, 2013), suggesting that SWRs are important for reactivation of experience related neuronal ensembles in the absence of related sensory input (Wilson and McNaughton, 1994; Ji and Wilson, 2007; Ego-Stengel and Wilson, 2010). Each SWR event activates a large number of hippocampal neurons (50,000 to 100,000) (Chrobak and Buzsáki, 1996; Csicsvari et al., 2000; Colgin, 2016) and the spike content of SWRs is temporally and spatially coordinated to replay fragments of sequential activation (Wilson and McNaughton, 1994; Nádasdy et al., 1999; Lee and Wilson, 2002; Pfeiffer and Foster, 2013). Neuronal activations during SWRs propagate to a large number of cortical and subcortical structures that are involved in the consolidation of episodic memory (Buzsáki et al., 1983; Wilson and McNaughton, 1994; Ji and Wilson, 2007; Jadhav et al., 2012). This reactivation is thus likely to represent a key component of the “two-step” hypothesis of memory consolidation (Buzsáki, 1989).

SWRs spontaneously occur in the hippocampus at a rate of 30–200 events per minute. Their abundance declines with age (Kanak et al., 2013; Buzsáki, 2015; Wiegand et al., 2016) and is vulnerable to hippocampal injury occurring with Alzheimer's disease and other neurodegenerative conditions (Hermann et al., 2009; Gillespie et al., 2016; Iaccarino et al., 2016; Nicole et al., 2016; Witton et al., 2016). Disruptions to SWRs occur in parallel with impaired hippocampus-dependent, episodic-spatial memory (Girardeau et al., 2009; Iaccarino et al., 2016; Witton et al., 2016), and the changes often occur before the pathology of neurodegeneration becomes apparent (Kanak et al., 2013). Reduced SWR abundance and quality (Gillespie et al., 2016; Iaccarino et al., 2016), may contribute to impaired consolidation of recent experience (Marshall et al., 2006; Axmacher et al., 2008; Eschenko et al., 2008).

In this report we explore to increase the abundance of SWRs by “pacing”, i.e., to initiate SWR events by low intensity electrical shocks given at a rate similar to the spontaneously occurring rate. Since SWRs are thought to arise from the firing of a small number or even a single CA3 pyramidal neuron (de la Prida et al., 2006; Jahnke et al., 2015; Bazelot et al., 2016), a low intensity stimulus may improve initiation without affecting the underlying grouping of neurons or assemblies that participate in a SWR event. We address three questions using our *in vitro* model: (1) Can low intensity stimuli reliably increase the abundance of SWRs? (2) Can weak stimuli reliably evoke SWRs, without LTD or other habituation effects that may follow from stimulation that is of low frequency or intensity? (3) Can highly diverse SWRs be initiated given that the external stimulus is repeatedly delivered in proximity to the same neurons? Our results show promising answers. Evoking SWRs requires much less energy than producing a detectable local field potential signal in CA1 stratum pyramidale. SWR abundance can be substantially increased (to the rate of pacing) especially when the abundance is compromised. The stimulus does not induce a stereotyped SWR event; evoked SWRs show similar diversity as spontaneous SWRs. In addition, the pacing stimulus is reliable for a long time without detectable reduction in efficacy. These results suggest that weak stimuli facilitate the spontaneous generation of SWRs, rather than directly activating the entire ensemble of neurons participating in SWR events. Pacing SWRs might be further developed into a new deep-brain stimulus technique for improving hippocampal dependent memory, with single stimulus pulses at a low frequency (~1 Hz). The pacing protocol is thus distinct from the concept of improving memory by exciting the peri-hippocampal cortex with multiple pulses at a higher frequency (e.g., 250 pulses at 50 Hz, Suthana et al., 2012; Kucewicz et al., 2018).

## Methods

### Slice preparation

Slice preparation P21-P33 male and female C57/Bl6 mice were used to prepare paired hippocampal hemi-slices in accordance with a protocol approved by the Institutional Animal Care and Use Committee at Georgetown University Medical Center. Following deep isoflurane anesthesia, animals were rapidly decapitated. The whole brain was subsequently removed and chilled in cold (0°C) sucrose-based artificial cerebrospinal fluid (sACSF) containing (in mM) 252 sucrose; 3 KCl; 2 CaCl_2_; 2 MgSO_4_; 1.25 NaH_2_PO_4_; 26 NaHCO_3_; 10 dextrose; bubbled with 95% O_2_, 5% CO_2_. Hippocampal slices (480 μm thick) were cut in horizontal sections from dorsal to ventral brain with a vibratome (Leica, VT1000S). Slices were incubated in ACSF for at least 2 h before each experiment. ACSF used for maintenance and recording contained (in mM) 132 NaCl; 3 KCl; 2 CaCl_2_; 2 MgSO_4_; 1.25 NaH_2_PO_4_; 26 NaHCO_3_; 10 dextrose; bubbled with 95% O_2_, 5% CO_2_ at 26°C. Slices were incubated for at least 120 min before being moved to the recording chamber.

### Local field potential (LFP) recording

**Figure 1:**
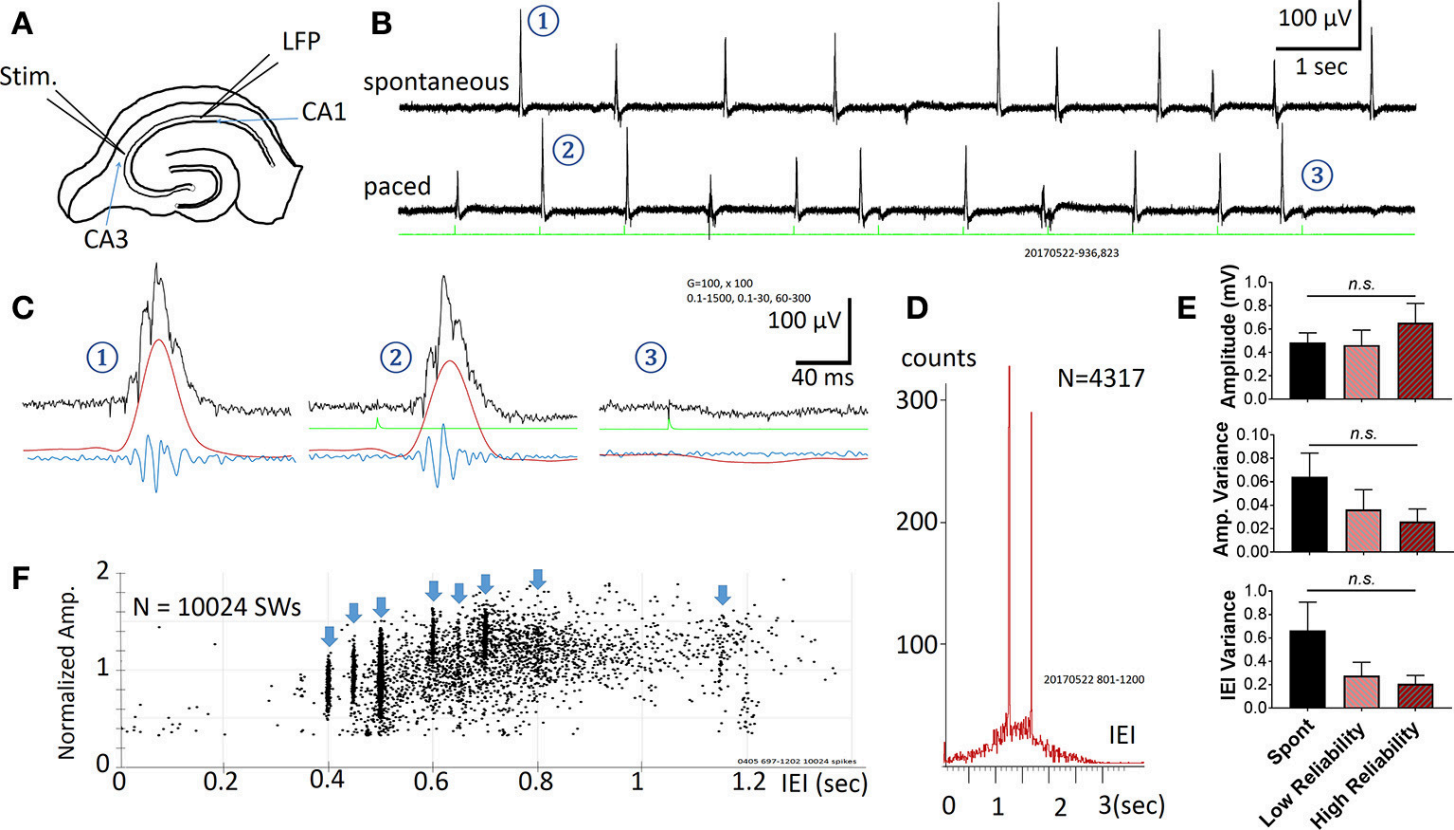
Spontaneous and Evoked SWRs. **(A)** Experimental preparation for stimulation and recording. **(B)** Spontaneous SWRs (upper trace), and evoked SWRs by pacing stimuli from the same slice. Green trace shows stimulation recorded in a hippocampal slice. Stimulus intensity in this example is 3.7 μA. **(C)** Examples (1-3 from the traces in **B**) on expanded time scale and different band pass filtering. Black = 0.1–1,500 Hz, Red = 0.1–30 Hz, Blue = 60–1,500 Hz. Note that 3 is a failed example, often occurring when a stimulus is delivered shortly after a spontaneous SWR. **(D)** Distribution histogram of spontaneous and evoked SWRs (*N* = 4317 SWRs). Pacing stimuli were delivered either at 0.8 Hz or 0.6 Hz, respectively faster and slower frequencies than the peak of the IEI distribution for spontaneous SWRs (~1.5 s or 0.67 Hz), forming two peaks in the IEI distribution histogram. **(E)** Comparison between amplitude, variance of amplitude, variance of inter-event interval (IEI) across 3 conditions: Spontaneous SWR events, low reliability evoked events, and high reliability evoked events. A low reliability stimulation is here defined as a success rate between 50 and 90%, whereas a high reliability stimulation is defined as a success rate >90%. The frequency of stimulation was chosen to be within a physiological range (within 150% of the spontaneous SWR rate). The stimulus intensity for all events considered here was significantly below the intensity needed to evoke a typical population spike (as seen in Figure 2). No significant differences were found (1-way ANOVA, multiple comparisons correction), although a trending increase in amplitude and decrease in variance was seen for higher reliability stimulations. *n* = 9 slices from 9 animals. Error bars are SEM. **(F)** Amplitude vs. IEI scatter plot from another preparation, pacing with multiple intervals (blue arrow heads). The amplitude of evoked SWRs showed large variability, within in the range of spontaneous SWRs, while the IEIs of evoked SWRs formed clusters surrounded the pacing interval.

LFP recordings were done in a submerged chamber, and slices were placed on a mesh that allowed perfusion on both sides at a high flow rate (10–30 ml /min) (Hájos et al., 2009; Maier et al., 2009). We use low resistance glass microelectrodes (~150 kΩ tip resistance). The electrodes were pulled with a Sutter P87 puller with 6 controlled pulls and filled with 0.5 M NaCl in 1% agar, which prevents leakage of the electrode solution that could potentially alter the tissue surrounding the electrode tip. The recording electrode was placed in CA1 stratum pyramidale where both sharp waves and ripples have large amplitudes (Maier et al., 2009). In each slice, several locations were tried to find an optimum recording location with high signal-to-noise (Figure 1). Usually the highest SWR peak was >100 μV and the ripple amplitude was 2–3 times higher than the noise level. In order to reduce the artifact of pacing stimulation, a reference electrode was placed outside the tissue, near the recording electrode. The location of the reference electrode was adjusted to achieve minimum stimulation artifact. The data were amplified 1000x with a custom made amplifier, filtered at 0.01–1,000 Hz and digitized at 3,000 Hz by a 12-bit USB Analog-to-digital converter (National instruments). From each brain slice we recorded 3–18 h of spontaneous and paced SWRs from the same recording site.

### Stimulation

Two types of pacing electrode, “focused” and “diffuse”, were used. A focused stimulus was provided by a single micro glass pipet with a 1–2 μm opening, placed into the tissue of CA3 stratum pyramidale. A diffuse stimulus was provided by a theta glass pipet (Warner Instruments TGC 150-10) with tip opening of 40–120 μm. The pipet opening was placed over CA3 stratum pyramidale without contacting the tissue. The distance from the tip opening to the tissue surface was about 30–60 μm, adjusted visually under stereo microscope magnification.

### Data analysis

SWR events were identified by threshold. The raw LFP traces were digitally filtered between 1 and 30 Hz, with a threshold set manually above the baseline noise to identify the majority of SWR events. Custom programs were written in Labview for digital filtering, threshold detection, and determining the amplitude and frequency distributions. Further analysis to determine stimulus success/failure rate and latency was conducted with custom algorithms written in MATLAB. A SWR was considered to be evoked if the SWR peak time fell within a window of +10 ms to +100 ms relative to stimulus onset. Outside of this window it was considered a spontaneous event. A similar though slightly less stringent window was used to classify a stimulus as a success or failure. A stimulus was considered a success if the peak time of a SWR event fell within a window of −10 to +100 ms relative to stimulus onset (as there is ~20 ms from SWR onset to peak, this window still resulted in stimulus preceding SWR). Otherwise the stimulus was considered a failure. The amplitude of SWR events was measured from the baseline to the peak of the 1–30 Hz filtered LFP.

### Statistics

Statistics were conducted in Graphpad Prism 7.0. To compare differences in mean we performed 1-way ANOVA with Tukey multiple comparisons correction. All error bars displayed are SEM. ^*^p < 0.05.

## Results

Spontaneous SWRs reliably occur in most hippocampal slices as reported by other groups (Kubota et al., 2003; Maier et al., 2003; Colgin et al., 2004; Behrens et al., 2005; Miyawaki et al., 2014; Keller et al., 2015). Electric stimuli were found to reliably evoke SWRs (in all 22 slices tested from 18 animals). A representative example is shown in Figure 1. Spontaneous and evoked SWRs (Figure 1B) both show variable amplitude and accompanying ripple oscillations (Figure 1C).

**Figure 2:**
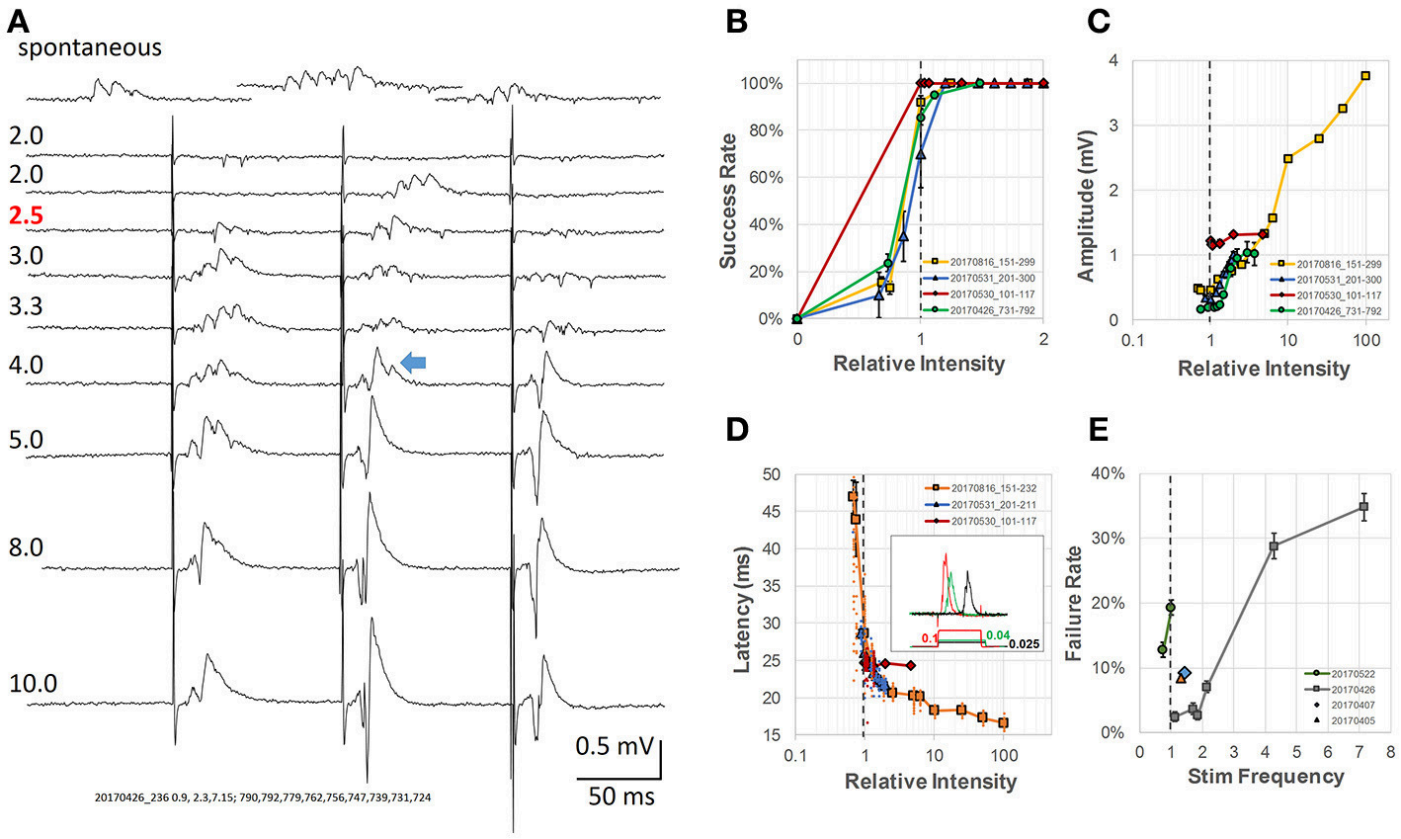
Analysis of intensity of pacing stimulus-response **(A)** Evoked response LFPs at different stimulus intensities. Numbers above each trace label relative intensity and actual current (μA, pulse duration 0.1 ms, red number indicates the threshold). *Top trace*, spontaneous SWRs from the same slice; The blue arrow indicates an example of a mixture of SWR and conventional LFP response (population spike). **(B)** Stimulus intensity vs. success rate. Threshold stimulus was defined as the lowest stimulation with at least 50% success rate of evoking SWRs (gray dashed line—threshold intensities normalized to 1 for four different animals). **(C)** Amplitude of evoked response vs. relative intensity, normalized to threshold for four different animals. **(D)** Latency between stimulus and SWR peak vs. relative intensity (normalized to threshold). Inset: examples of wide stimulus pulses at even lower stimulus intensity (0.1, 0.04, and 0.025 of the relative threshold intensity at 0.1 ms pulse). **(E)** Rate of pacing failure vs. pacing frequency. Different preparations normalized to their spontaneous SWR rate, broken line shows the normalized spontaneous frequency for four animals. All error bars are SEM for the individual condition and animal.

**Figure 3:**
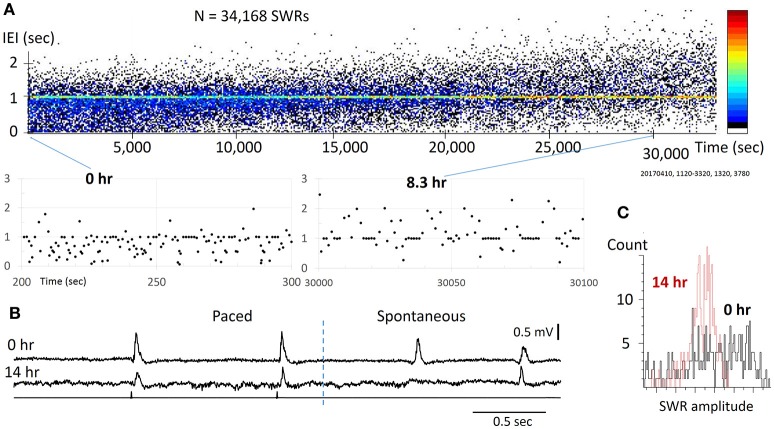
Reliability for pacing SWRs. **(A)** top, scatter plot of IEI vs recording time. Note that the IEI increases gradually over time (hours), apparently caused by the decline of slice viability. bottom, two sections from the top plot with expanded time scale, showing that stimulation can reliably evoke SWRs through the course of the experiment. The pacing rate was 1 Hz, eight pacing stimuli were given as a train, intermitted with a 7-s period with no pacing. The pseudo-color codes to the right indicates SWR density (from cold to warm colors) that increased with time. **(B)** Example traces from the slice at 0 h (top) and 14 hours (bottom) of the recording time, showing significance decline in amplitude, but the pacing remains reliable. **(C)** The SW amplitude decreased and its distribution changed largely over time, indicating reduced diversity of spontaneous SWRs.

The pacing stimuli often failed to evoke a SWR when a spontaneous SWR occurred shortly before the stimulus (two failed events are seen in Figure 1B). When evoked SWR failures were observed, a small “monophasic potential” was induced (Figures 1C–**3**), likely to be a subthreshold synaptic event.

The occurrence rate of SWRs is quantified as the Inter-Event-Intervals (IEIs) in this report. Figure 1D shows the IEI distribution histogram from 4317 spontaneous and evoked SWRs recorded from a single slice. The IEIs of spontaneous SWRs were highly variable, distributed in a range between 0.1 and 3 s, with a peak approximately at 1.5 s in this example. The pacing stimuli were given at two intervals, 1.25 s (0.8 Hz, for 1,500 s) and then 1.67 s (0.6 Hz for 1,500 s), intermitted by 1,500 s of recordings with no pacing. These two pacing rates were given near the peak of the IEI distribution of spontaneous SWRs, and both reliably evoked SWRs. The paced SWRs form sharp peaks in the IEI distribution histogram, in contrast to the wide distribution of spontaneous SWRs. Across 9 animals, we observed no significant change in amplitude, amplitude variance, and IEI variance between spontaneous and low-intensity evoked SWRs (Figure 1E).

We tested a variety of pacing frequencies, faster or slower than the peak of spontaneous IEI, and all showed reliable pacing of SWRs (Figure 1F). These results suggest that evoked SWRs, like spontaneous ones, can occur in a wide range of frequencies, and that pacing at a faster rate than the average spontaneous rate can reliably generate more SWRs. The evoked SWRs also displayed highly variable amplitude. The eight pacing frequencies tested in the example in Figure 1F formed eight vertical clusters formed in the amplitude-IEI graph, demonstrating a highly variable SWR amplitude evoked by the same pacing stimulus, regardless of the pacing frequency. The amplitude variability of evoked SWRs fall in the same range of spontaneous ones, suggesting the pacing stimulus is able to evoke diverse SWRs similar to spontaneous SWRs. The amplitude of SWRs reflects the total number of active neurons participating, suggesting the same stimulating intensity and pacing frequency can evoke highly variable SWRs, as quantified in Figure 1E.

Stimuli that failed to evoke a SWR generated a small monophasic potential, instead of the conventional population spike (PS) LFP response in the CA1 area (Figure 1C). This suggests that the stimulus intensity required for evoking SWRs may be smaller than that needed to evoke a PS. We used a wide range of stimulus intensity to examine the threshold required to evoke a SWR and to distinguish from conventional evoked PSs. In the example traces shown in Figure 2, we used 10 Hz pulses as stimuli for the test. At this stimulating frequency, paired-pulse facilitation can be used for identifying near threshold intensity. In addition, shorter stimulus intervals would allow SWRs to be evoked only by the first few stimuli; stimuli pulses later in the train would fail to evoke SWRs but can still evoke PSs.

Below threshold, trains of single pulses (0.1 ms wide) failed to evoke any response (Figure 2A, traces 1–2). Increasing the stimulus current, the second pulse of the train (Figure 2A, trace 3), often evoked SWRs, suggesting that while the intensity is still sub-threshold for the first pulse, the second pulse becomes an effective stimulus due to paired-pulse facilitation. Further increasing the stimulus current resulted in reliably evoked SWRs for the first few pulses of the train (Figure 2A, traces 4–5). The evoked SWRs have similar amplitude and ripple cycles to spontaneous SWRs observed in the same slice (Figure 2A, top).

Further increasing the intensity resulted in a mixed response of SWRs and conventional PSs, which displayed higher amplitude than SWRs but with a reduced ripple oscillation (Figure 2A, traces 6–7). SWRs and conventional PSs could be easily distinguished. SWRs appeared as an all-or-none oscillatory response, having similar amplitude and frequency regardless the stimulus intensity. In contrast, conventional PSs were characterized by a tri-phase peak and amplitude that increased with stimulus intensity as classically reported (Bliss and Gardner-Medwin, 1973).

At even higher stimulus intensity, ripple oscillations disappeared, while only conventional PSs were observed (Figure 2A, traces 8–9). We next examined in more detail the threshold intensity, latency, and success rate at different stimulus intensities and pacing frequencies. The stimulus intensity for evoking SWRs was consistently observed to be much smaller than that for evoking conventional PSs in all slices tested. We defined the threshold as the stimulus for which the success rate of evoking SWRs was ≥ 50%, and employed a relative intensity scale with this threshold set to 1. Using this relative intensity scale, we noticed the success rate for evoking SWRs quickly increased to near 100% (Figure 2B). The stimulus intensity and the amplitude of conventional PSs displayed a positive correlation across a large intensity range (Figure 2C). The amplitude of spontaneous and evoked SWRs were 50-500 μV, much smaller than the evoked PS that might reach 3 - 4 mV at high intensity stimulus (yellow curve in Figure 2C). A mixture of SWR and evoked PS (blue arrow in Figure 2A) likely accounts for the amplitude increase in the intensity range between 1 and 10 of the threshold.

We also observed that the latency between stimulus onset and SWR peak quickly changed from highly variable to a stable ~20 ms (Figure 2D). To further verify that weak stimulation was effective, we used a longer duration stimulus pulse (150 ms) at even lower intensity (Figure 2D inset). To our surprise, a longer stimulus pulse at sub-threshold intensity could still evoke SWRs, although with lower probability and longer latency. This lower-intensity and longer-duration stimulus is likely to induce a prolonged depolarization in many neurons, as opposed to the supra-threshold shorter-duration stimulus. To test the limits for how fast SWRs could be paced, we increased the stimulation frequency to up to 7 times the physiological spontaneous SWR rate, and found that although the failure rate increased for the animals tested, a failure rate below 50% could still be achieved (Figure 2E).

These results are consistent with the findings of Bazelot et al. (2016), that very weak stimulus, activating only a single CA3 pyramidal neuron is capable of evoking a SWR event. Stronger and synchronized CA3 pyramidal neuron activation, in contrast, would abolish SWRs, as suggested by Ego-Stengel and Wilson (2010).

Weak stimuli were also effective with a diffuse stimulation, i.e., when the stimulation electrode was not in contact with the tissue. In this non-contact stimulation, a large number of neurons are more evenly affected by the stimulus. Diffuse stimulation also allows for reducing the effects of electrochemical reactions and local damage to the stimulation site. In this report, diffuse stimulation was used for most long-term recordings (e.g., Figure 3).

### Evoked SWR reliability

It is unknown whether repetitive weak stimuli would decrease the reliability for pacing SWRs, given that long-term depression (Dunwiddie and Lynch, 1978) or other habituation effects in the circuit may develop after repetitive ~1 Hz stimulation pulses.

We tested the reliability of weak stimuli to evoke SWRs on a time scale of 10–20 h, which was approximately the longest that acute brain slices could be functionally maintained *in vitro*. Within this period, tens of thousands of stimuli are given. In the example shown (Figure 3A), SWRs were paced at 1 Hz for 14 h, during which 26,880 identical stimulus pulses were given onto the same CA3 population. We found the stimulation was reliable even toward the end of the recording session (Figure 3B). For long-term recordings, diffuse (non-contact) stimulation was used to reduce mechanical and electro-chemical damage near the stimulating site. We tested the pacing reliability overnight in 6 slices from 6 distinct animals, all of which showed reliable pacing for over 10 h. The longest test was 20 h of continuous pacing (28 h after slicing). Possibly due to variations in the viability of the preparation, spontaneous SWRs changed with time. The pacing rate was initially set to produce a higher rate of spontaneous SWR, but as the spontaneous SWR rate decreased with time, associated perhaps to cell loss, there was a shift to a higher paced than spontaneous rate. The trend of shifting from spontaneous to evoked SWRs can be seen as an increase in the IEI (Figure 3A), where the pseudo-color codes the increase in SWR density (from cold to warm colors). In addition, we observed a reduction in amplitude and skewing of its distribution (Figure 3C). Despite this decline, stimulation could still reliably evoke SWRs, suggesting that pacing can be highly reliable even in a damaged network. These results suggest that weak pacing stimulus does not induce detectable LTD or habituation in the initiation of SWRs.

### Restoring SWR abundance

We next tested the pacing protocol in slices in which the abundance of spontaneous SWRs was acutely compromised. It is known that GABAergic local inhibitory neurons are important for generating SWRs (Schlingloff et al., 2014; Stark et al., 2014), and that low concentrations of GABA antagonists can compromise SWR abundance (Ellender et al., 2010). Considering this, we tested if reducing GABAergic inhibition resulted in changes to the rate of spontaneous SWRs, and whether pacing stimuli restored normal SWR abundance.

**Figure 4:**
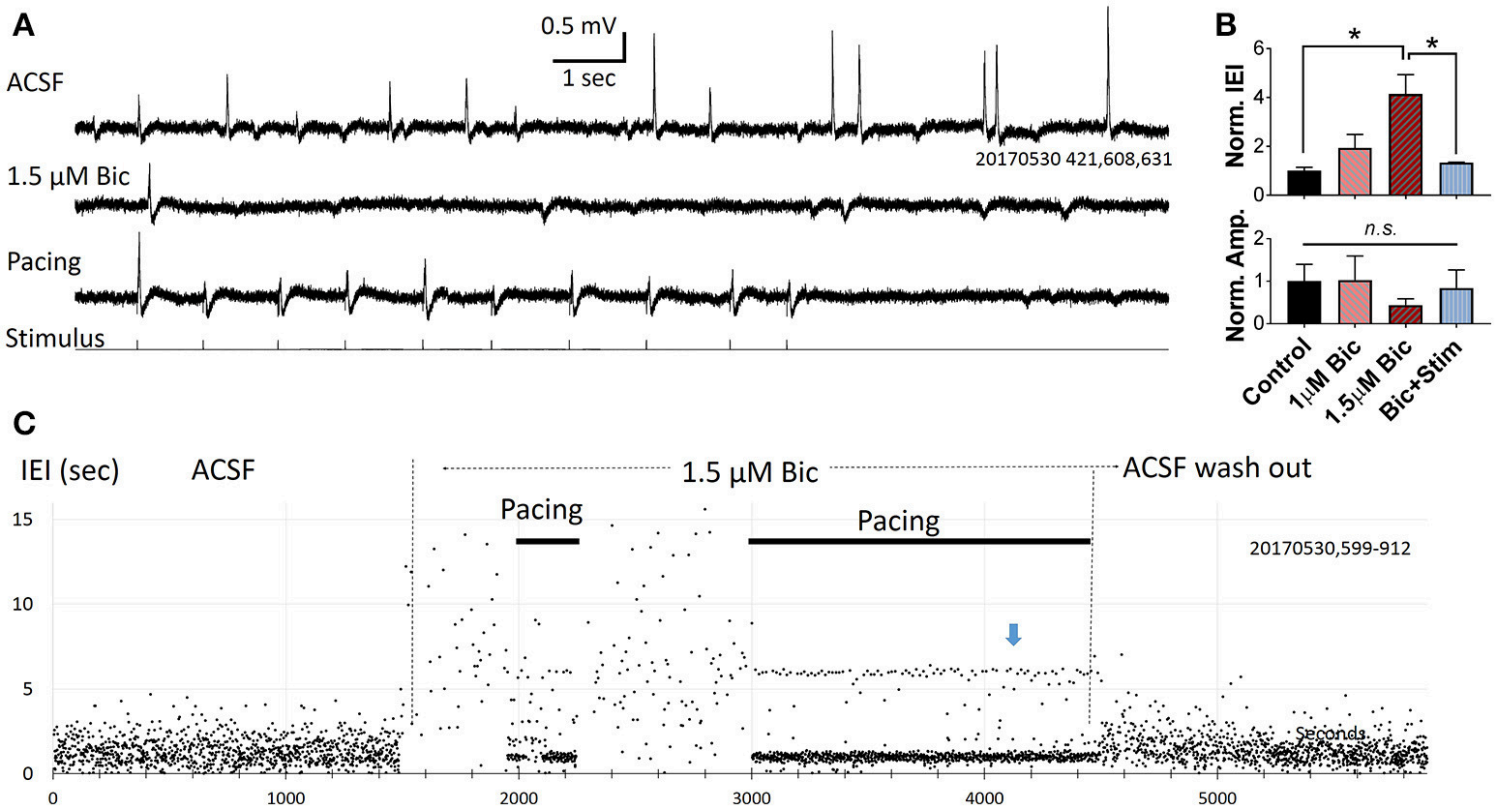
Pacing compromised SWRs. **(A)** Recording traces from a slice bathed in ACSF (top), 1.5 μM bicuculline without pacing (middle) and with pacing (bottom). In each consecutive 15 s recording trial we give 10 pacing pulses and 5 s with no pacing. The rate of pacing is 1 Hz with 20% randomness in intervals. **(B)** Plots from three slices showing changes in relative IEI (top), and amplitude (bottom), in bicuculline. IEI and amplitude are normalized to the average control condition. *n* = 3 animals, 1-way ANOVA, multiple comparisons correction. *p < 0.05. Error bars are SEM. **(C)** IEI affected by bicuculline and pacing. Note that the effect of bicuculline is reversible. Blue arrow indicates an IEI cluster of 6 s, formed by the time interval from last pacing pulse in each trial and the first pacing pulse of the next trial. Indicating that there was no spontaneous SWRs when pacing was turned off.

In three slices we applied a low concentration of bicuculline (1–2 μM) via bath application, and the abundance of spontaneous SWRs observed was greatly reduced (Figure 4A). Significant changes were seen when bicuculline concentration was increased to 1.5 μM (Figure 4B), demonstrating that the spontaneous SWR rate and amplitude are highly sensitive to GABA blockade. Under bicuculline administration, pacing can still reliably evoke SWRs (Figure 4A, bottom trace). SWR abundance can be fully restored when the pacing rate was adjusted to the spontaneous rate observed prior to bicuculline administration (Figure 4B,C). In the example shown in Figure 4C, under 1.5 μM bicuculline, the rate of spontaneous SWR is largely reduced (resulting in a higher IEI). The LFP signal is recorded in consecutive 15 sec recording trials. In each trial we give a train of 10 stimuli (1 Hz, 20% randomized interval) and then wait ~6 s with no stimulus (bottom trace of Figure 4A). If there were no spontaneous SWRs during this no-stimulus period, a ~6 s IEI is generated, between the end of the last pacing pulse of trial *n* to the first pacing pulse of trial *n*+*1*. Over consecutive recording trials an IEI cluster of 6 s is generated (blue arrow in Figure 4C). This cluster, in addition to the larger cluster around the pacing frequency, is a strong indicator that most SWRs are evoked when the GABAergic inhibition is compromised.

### Stimulation with randomized intervals

**Figure 5:**
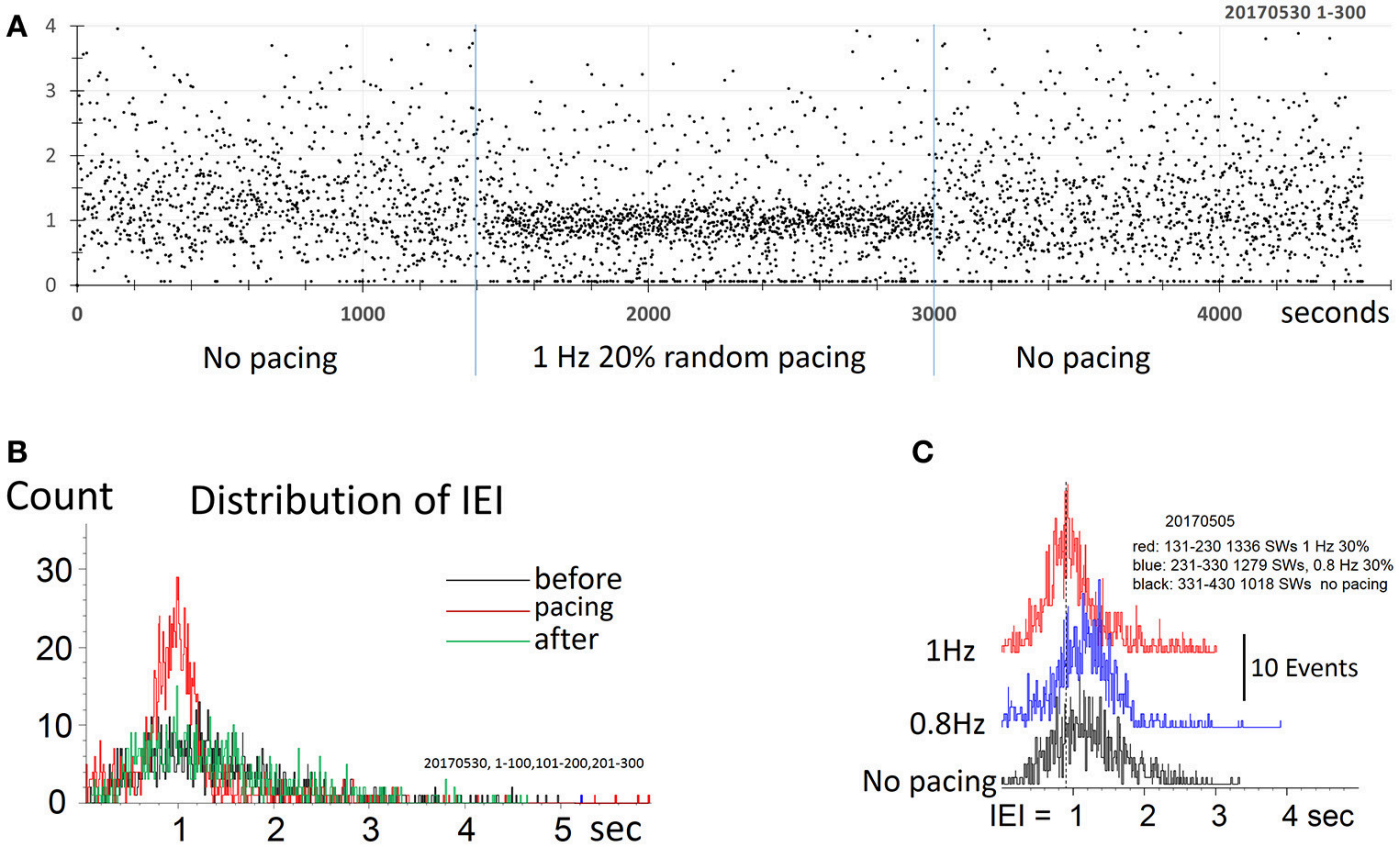
Pacing SWR by randomized stimulus intervals. **(A)** Pacing by randomized intervals (two blue lines mark duration of stimuli) generates more frequent SWRs (shorter IEI) around 1 Hz, with 20% randomized timing. **(B)** density distribution of IEI, demonstrating that randomized pacing increased the abundance of SWR around the spontaneous peak of IEI distribution. **(C)** Density distribution of IEI, with randomized pacing in two center frequencies, faster and slower than the spontaneous IEI rate. Note that both faster and slower pacing can increase the abundance of the SWRs.

As the IEI distribution was notably different between paced and spontaneous SWRs, with spontaneous events displaying a broad distribution in IEIs and paced events occupying a narrow distribution (Figure 1D), we next tested whether introducing a random pacing frequency altered the IEI distribution of evoked SWRs. We set the stimulus intervals in a 20–30% random range from a central value. The effect of pacing can still be clearly observed with a randomized frequency (Figure 5A). Randomized pacing stimuli generated SWRs with a uniform distribution peak around the central value of 1 Hz (Figure 5B), eliminating the fixed IEI peaks seen with a set stimulus intervals (Figure 1D). Given that evoked SWRs can reach at least 2 times the spontaneous frequency with low failure rate (Figure 2E), setting the randomized pacing rate higher and lower than the spontaneous rate can still reliably evoke SWRs with variable intervals (Figure 5C).

## Discussion

The main findings of this report are that SWRs can be evoked by low intensity electrical stimulation, and at lower intensities than those needed to evoke detectable population spikes. In addition, evoked SWRs show large variability in amplitude, similar to that of spontaneous events, despite pacing stimuli having the same intensity and being applied to the same location. Moreover, low intensity pacing at ~1 Hz can reliably evoke thousands of SWRs without detectable LTD or habituation as seen in hippocampus (Bliss et al., 2007; Pinar et al., 2017). Pacing can effectively increase the abundance of SWRs, especially when the spontaneous SWR rate is compromised. These results suggest that the stimulus is not directly exciting the majority of neurons participating in the SWR event; but rather that the pacing stimulation facilitates the emergence of spontaneous SWR events.

### Weak vs. strong stimulus

Weak stimulation, which may activate a single CA3 neuron, is capable of initiating a SWR event (Bazelot et al., 2016). However, our weak stimulation differs from that of Bazelot et al., in that it does not involve a supra-threshold activation of a few cells. Instead, our stimulation is more likely to represent a small subthreshold depolarization in a large population of neurons. In a large portion of our experiments the stimulating electrode is not in direct contact with the tissue, and thus many neurons in similar proximity to the electrode may be equally depolarized. This sort of diffuse stimulation is likely to produce sub-threshold depolarizations which may in turn increase the spontaneous firing rate of the CA3 population through lowering of the spike threshold. Such de-synchronized spikes are still capable of initiating SWR events with high efficacy (Figure 2B) but with variable latency (Figure 2D). Higher intensity stimuli induce synchronized spiking in the CA3 population and evoke detectable population spikes in the CA1 population, but with disrupted ripples (Figure 2A, bottom traces). In behaving animals, stronger hippocampal stimulation may disrupt both SWRs and memory consolidation (Ego-Stengel and Wilson, 2010).

Our results suggest that SWRs are self-organized events emerging from asynchronous firing in CA3. Weak electric stimulation may provide a background of elevated spontaneous firing, promoting the initiation of spontaneous SWRs.

### Why there is no detectable LTD/habituation?

We observed no detectable long term depression (LTD) or habituation of SWR after several thousand stimulus pulses at 1 Hz for over 10 h. Repetitive low intensity stimulation is known to induce LTD of population responses (Bliss et al., 2007). Why then did we not observe LTD or any reduction in the efficacy of evoking SWRs? One possibility is that our stimulus is too weak to produce consistently large EPSPs, insignificant compared to those underlying spontaneous spiking activity in CA3. Low frequency, NMDA receptor activating EPSPs are required to precipitate LTD. Spontaneous generation of SWRs has been shown to resist degradation over long periods without sensory input (Buzsáki et al., 1983), suggesting a robust process for generating spontaneous SWRs, resistant to LTD and habituation. Our pacing stimulation may facilitate the spontaneous initiation of the SWRs without altering the robustness of initiation process.

An additional possibility is that the action potential firing caused by weak electrical stimulation is highly variable between cells. Even with identical stimulus-induced depolarizations of CA3 neurons, the synaptic pathways in the population underlying the spiking sequence may be different from stimulus to stimulus. Highly variable synaptic pathways would not allow a determined pattern of spike timing dependent plasticity to form within the population. As suggested by Colgin et al. (2004), spontaneous SWRs actually impair long term potentiation (LTP) in hippocampus.

### Variations in evoked SWRs

The IEI and amplitude of both spontaneous and evoked SWRs are highly variable (Figure 1E). The amplitude of SWRs reflects the fraction of neurons involved in each event. Highly variable amplitude suggests a diverse participating cell membership in each event. We often observed low-amplitude evoked SWRs occurring immediately after a spontaneous SWR, suggesting the variability is partly due to coincidence with previous events. However, coincidence cannot fully explain the amplitude variability. When pacing at a higher rate, most of the SWRs observed are evoked rather than spontaneous. These evoked SWRs are not intermitted by spontaneous events but still display large variability in amplitude, suggesting an intrinsic characteristic of the network.

### Pacing rate

We show that the SWRs can be paced at a higher occurrence rate, often 2–3 times higher than the average of the spontaneous occurring rate (Figure 2E). This potentially permits pacing as a method for restoring the abundance of SWRs in the setting of hippocampal damage. We tested two situations in which the abundance of spontaneous SWRs is reduced (Figures 3, 4). Under both conditions, pacing was able to evoke a greater abundance of SWRs, potentially serving as a compensatory mechanism. While pacing could not improve the amplitude reductions observed over 12+ h of recording, a higher occurrence rate may permit relay, and could be beneficial for memory consolidation. The utility of this approach remains to be tested in aged animals or in animal models of neurodegeneration, in which the spontaneous SWR rate is compromised (Nicole et al., 2016; Wiegand et al., 2016; Witton et al., 2016). As a potential application, the pacing rate may be adjusted on demand, delivering stimulation only when there are insufficient spontaneous SWRs, with a stimulation rate set to an expected healthy spontaneous rate. In this report we have also tested a randomized pacing rate for evoking SWRs (Figure 5). The IEI distribution of spontaneous SWRs fits a lognormal distribution (Buzsáki, 2015). Adding a randomized pacing could permit more physiological SWRs to be induced and avoid fixed intervals of replay.

### Possible mechanism

**Figure 6:**
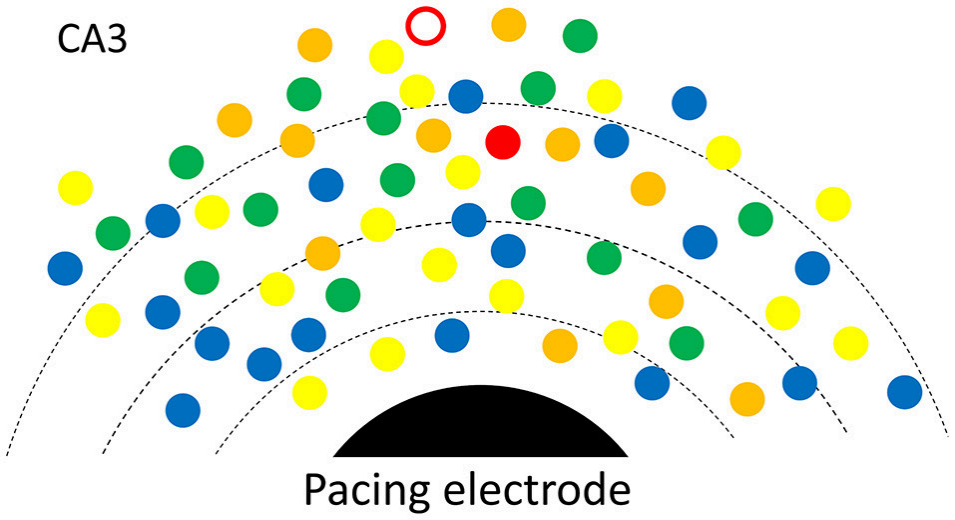
Possible mechanism. A SWR is initiated from spontaneously rhythmic (color dots) or rhythmic configurations of neurons (triangles). Multiple potential initiators are in different phases (colors). The electric field (broken lines) around the pacing electrode (black) would help the one with most advanced phase to win and to become the initiator for the next SWR. For each SWR event, the initiator (red open circle or the red triangle) is not necessarily closest to the electrode.

To better explain how low intensity pacing is both effective and reliable, we propose a possible mechanism in which multiple potential initiators are involved in the initiation of a SWR event (Figure 6). An initiator may be composed of a small group of spontaneously active neurons, as suggested by Jahnke et al. (2015), de la Prida et al. (2006) and Bazelot et al. (2016). There may be a competition between potential initiators with the winner initiating the next SWR event. The reliability of the weak pacing stimulus would be high because the stimulus affects many potential initiators concurrently and the cumulative probability over all initiators is high. This is consistent with the finding that a longer duration stimulation pulse (150 ms) can evoke SWRs with only 10% of the intensity threshold for shorter stimuli (Figure 2D, inset). A longer duration pulse would increase the cumulative probability of a greater number of potential initiators than a shorter pulse, resulting in a successful SWR initiation. Given that the stimulus produces a small depolarization in many neurons, the winner is not necessarily located closest to the electrode (red circle in Figure 6). Multiple competing initiators may also explain why the same pacing stimulus could initiate SWRs with large variability in amplitude (Figure 1E), as the initiator for each SWR is a consequence of competition, instead of those closest to the stimulating electrode.

Initiation of SWRs requires a process of great amplification, from scattered spontaneous activity to organized population activity involving a large numbers of neurons (Buzsáki, 2015). Our pacing stimulus seems to provide an initial push, which may directly induce spontaneous firing, or advance the phase of rhythmically active neurons. In this sense our low intensity stimulation is in fact “evoking a spontaneous activity” (Petersen, 2005).

Whether experience relevant replay is increased with evoked SWRs and more importantly, whether this can enhance memory consolidation remains to be studied *in vivo*. If so, weak and diffuse electric stimuli could be adopted by non-invasive therapeutic applications including tDCS (Reato et al., 2010; Xu et al., 2014) or TMS.

## Author contributions

HJ and SL contributed equally; JW contributed conception and designed the study; HJ, SL and JW conducted the experiments; AC and XG performed the statistical analysis; JW wrote the first draft of the manuscript; XG, AC, KC, and SV wrote sections of the manuscript. All authors contributed to manuscript revision, read and approved the submitted version.

### Conflict of interest statement

The authors declare that the research was conducted in the absence of any commercial or financial relationships that could be construed as a potential conflict of interest.
